# Supplementary figures and images for: Spatio-temporal patterns of whiting (Merlangius merlangus) in the Adriatic Sea under environmental forcing
Source: PLoS One. 2024 Mar 22;19(3):e0289999. doi: 10.1371/journal.pone.0289999 (PMC10959384; doi:10.1371/journal.pone.0289999)

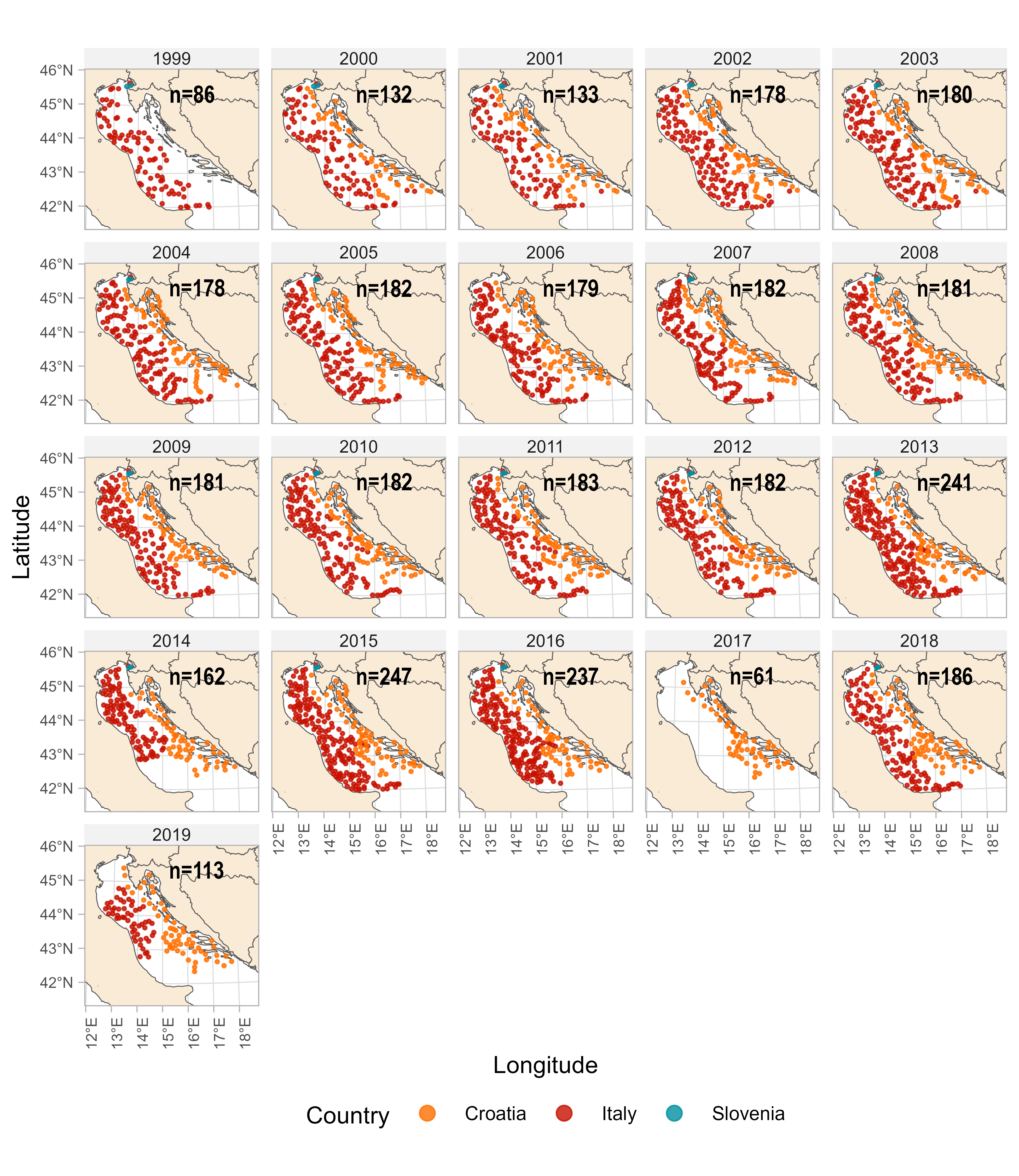

Supplement: S1 Fig — The number of hauls conducted per year is reported on the top right of each panel. Missing hauls (e.g., in 2014, 2017, 2019) were conducted in autumn-winter period, thus removed from the analyses to obtain a seasonally homogeneous dataset. In 1999 Croatia did not take part in the data collection. Made with Natural Earth. (TIF) [file pone.0289999.s001.tif]

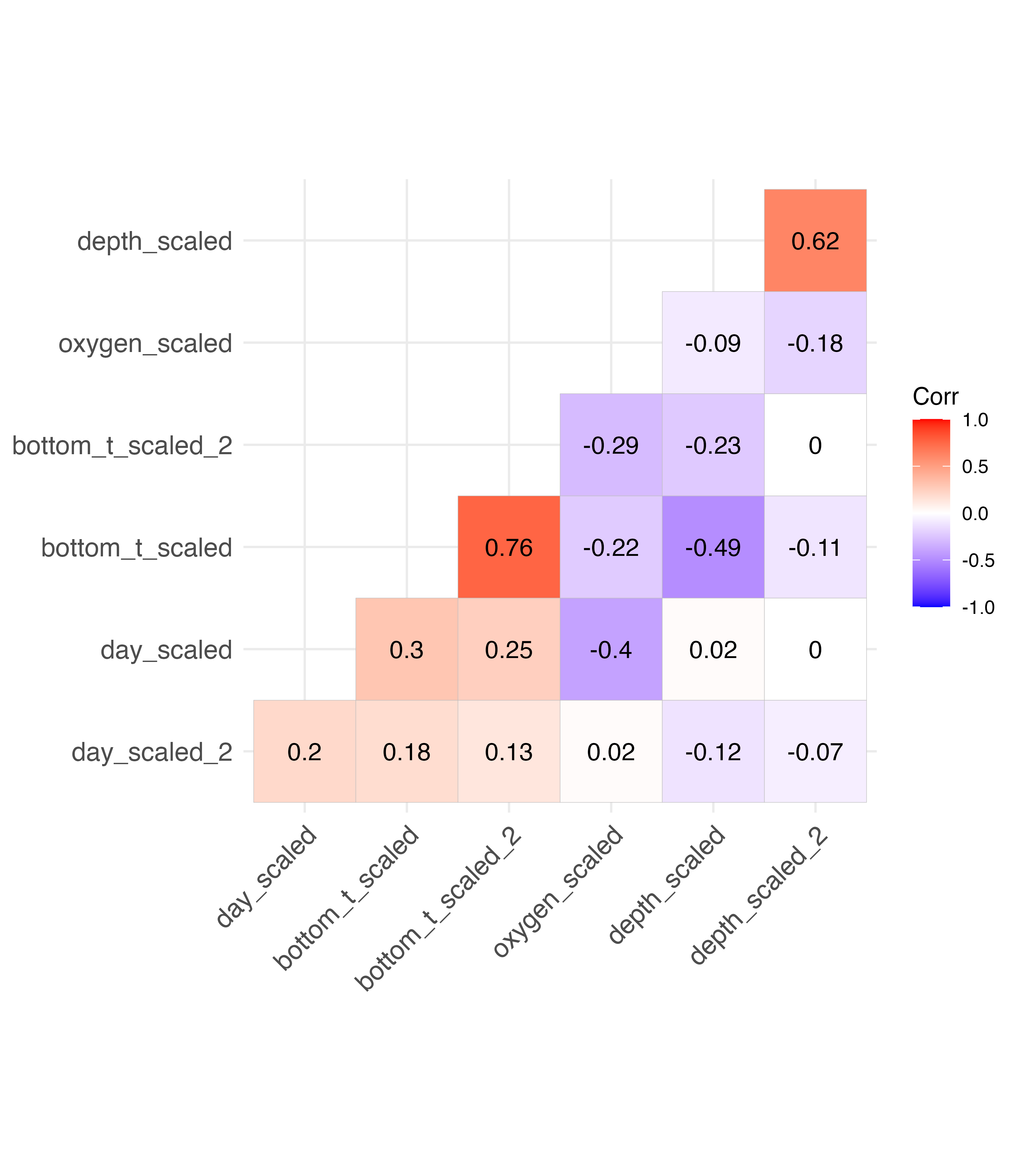

Supplement: S2 Fig — (TIFF) [file pone.0289999.s002.tiff]

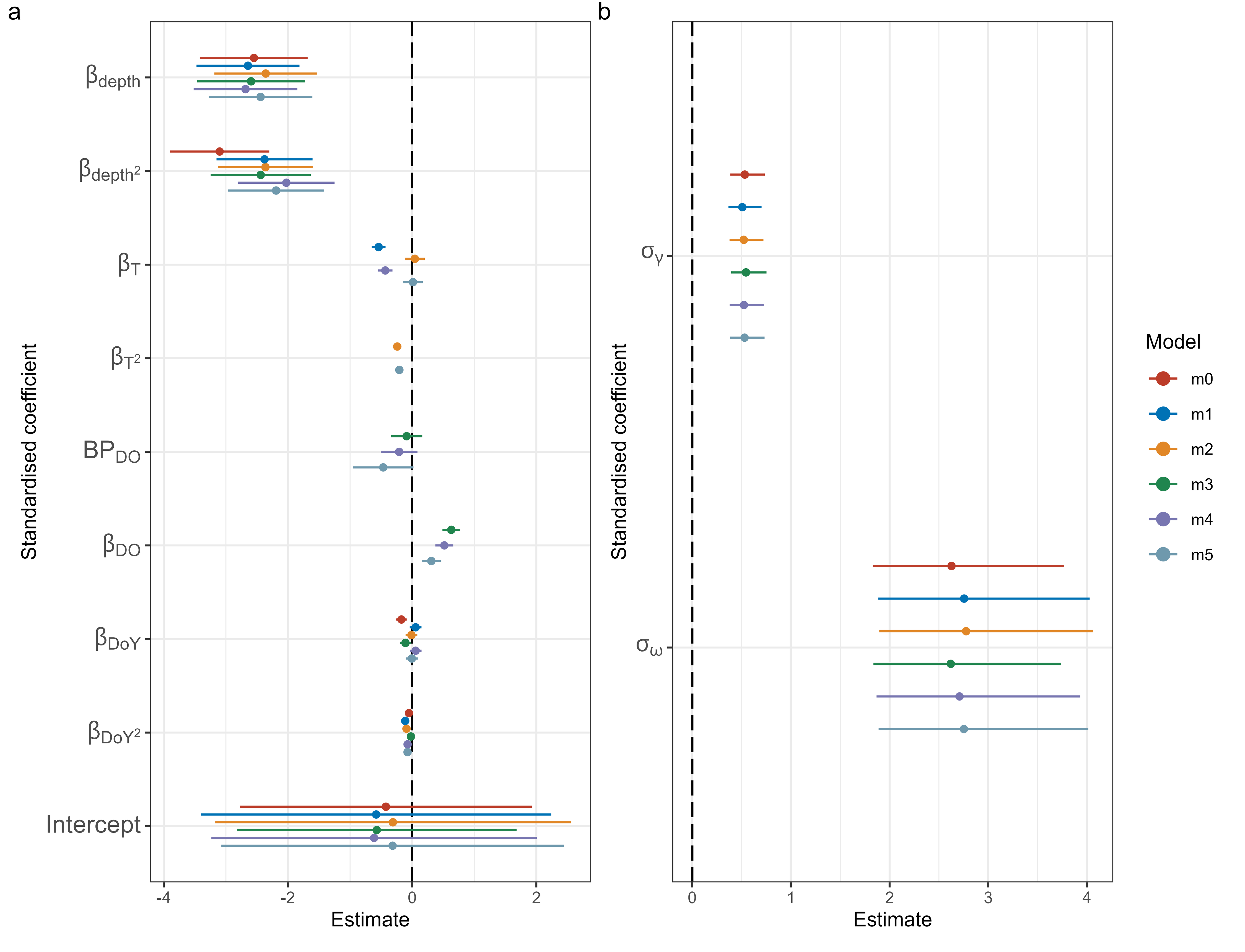

Supplement: S3 Fig — The point indicates the mean while the lines represent the 95% confidence intervals for the fixed effects (a) and the random effects (b). βDO represents the coefficient of the slope related to DO while BPDO represents the estimated breakpoint. σγ represent the standard deviation of the random year intercept and σω the standard deviation of the spatial random field. (TIF) [file pone.0289999.s003.tif]

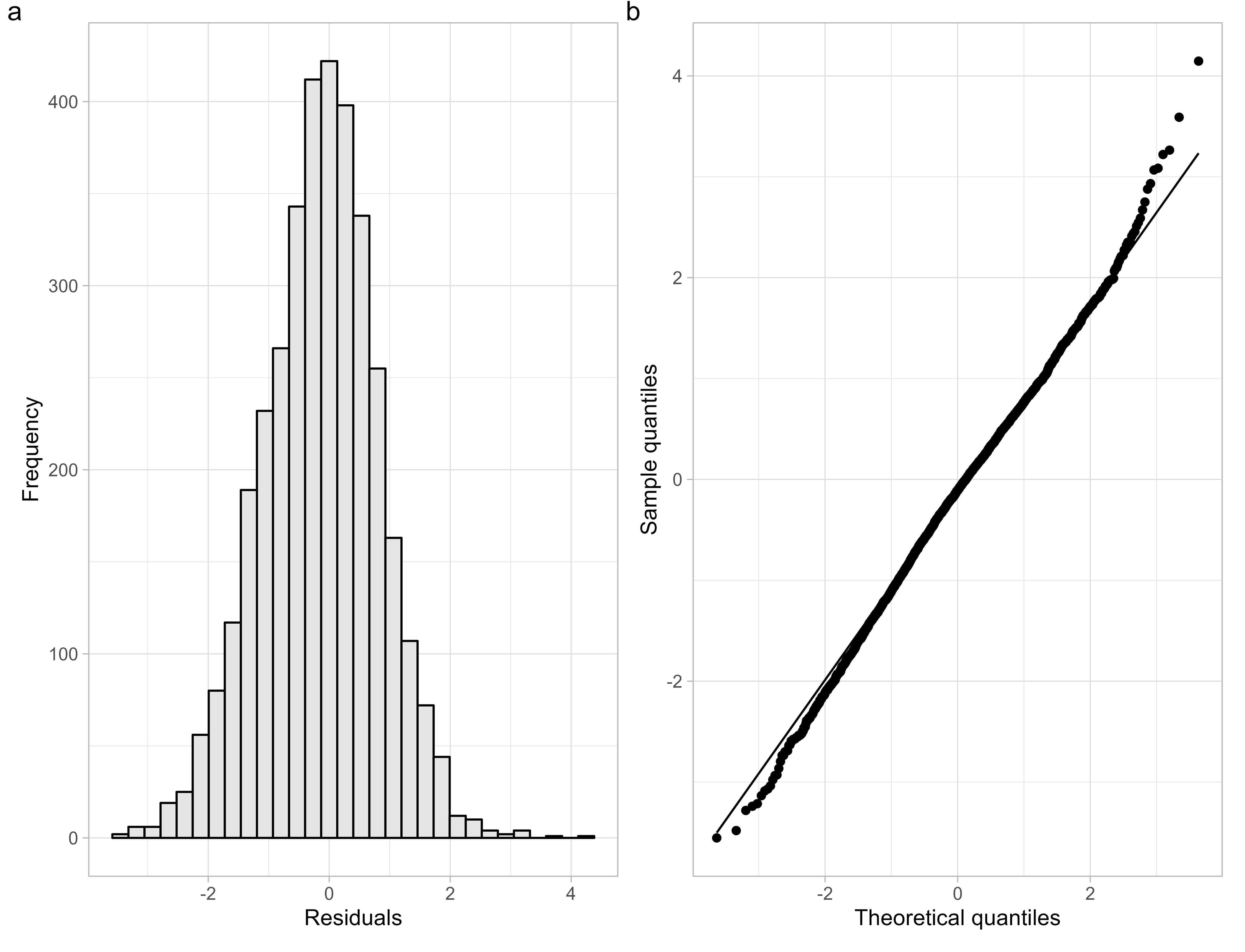

Supplement: S4 Fig — Histogram of randomized quantile residuals (a) and residual quantile-quantile plot (b). (TIF) [file pone.0289999.s004.tif]

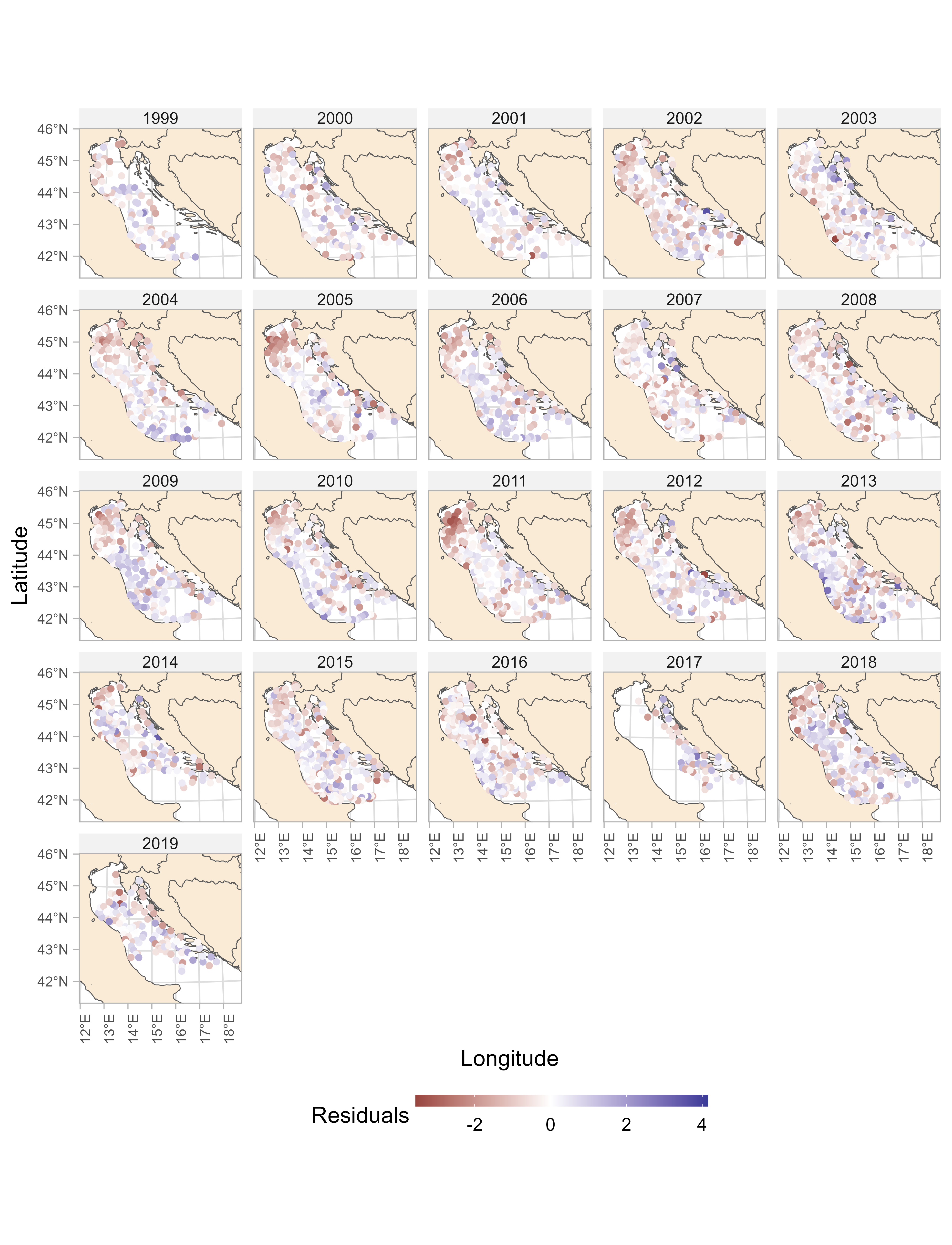

Supplement: S5 Fig — Made with Natural Earth. (TIF) [file pone.0289999.s005.tif]

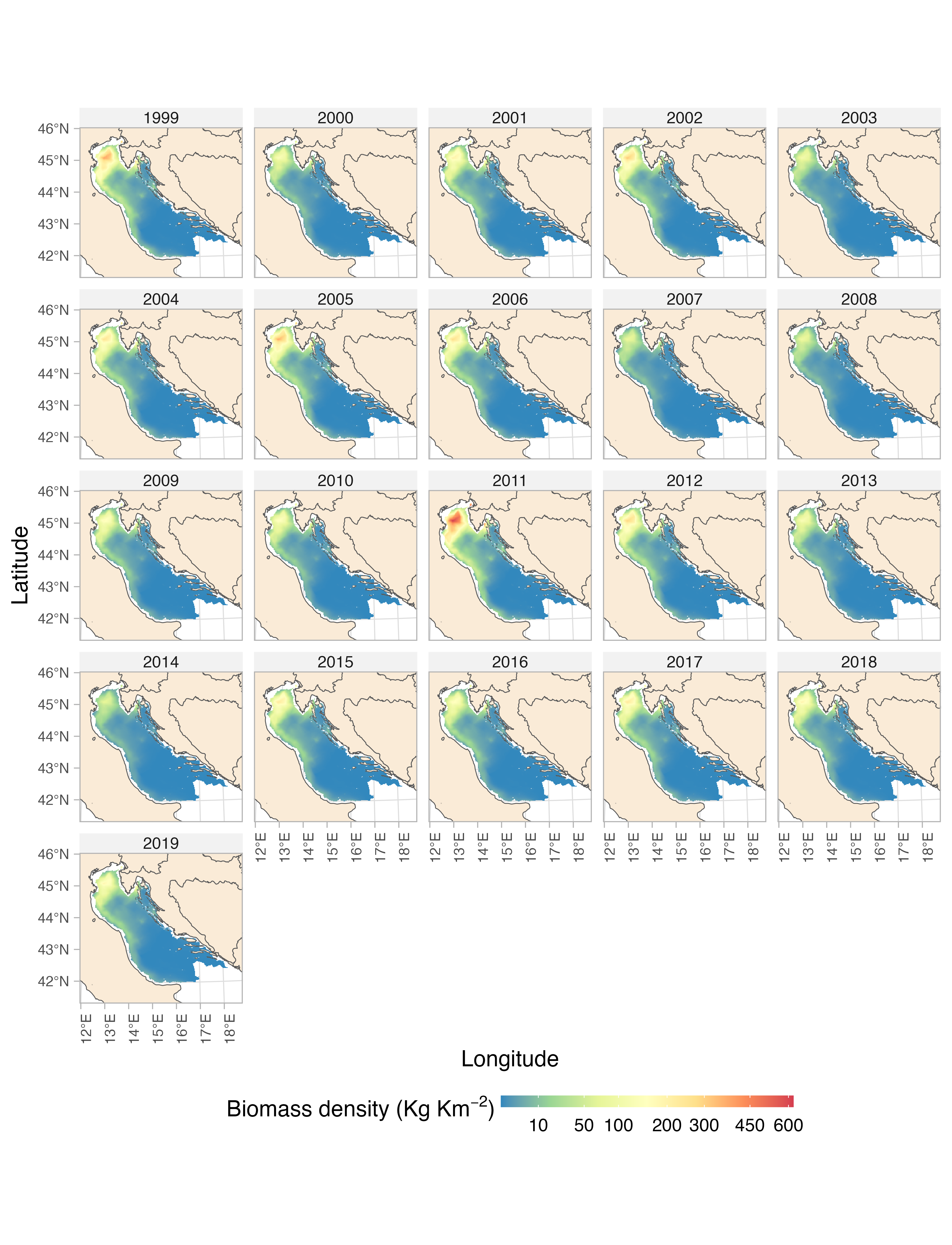

Supplement: S6 Fig — All predictions were made over a 2 km2 grid. Made with Natural Earth. (TIF) [file pone.0289999.s006.tif]
